# First report on molecular breast cancer subtypes and their clinico-pathological characteristics in Eastern Morocco: series of 2260 cases

**DOI:** 10.1186/s12905-016-0361-z

**Published:** 2017-01-09

**Authors:** Manal Elidrissi Errahhali, Mounia Elidrissi Errahhali, Meryem Ouarzane, Tijani El Harroudi, Said Afqir, Mohammed Bellaoui

**Affiliations:** 1 Medical Biology Unit, Faculty of Medicine and Pharmacy of Oujda, University Mohammed the First, Oujda, Morocco; 2 Hassan II Regional Oncology Center, Oujda, Morocco

**Keywords:** Breast cancer, Molecular subtypes, Prognosis factors, Morocco

## Abstract

**Background:**

Breast cancer is the most frequent malignancy among women in Eastern Morocco. In this paper, we provide the first report on molecular breast cancer subtypes in this region. This is the largest population–based study on breast cancer among Moroccan women.

**Methods:**

We analyzed 2260 breast cancer cases diagnosed at the Hassan II Regional Oncology Center between October 2005 and December 2012. Clinico-pathological and therapeutic features were studied. Molecular subtypes were determined and their associations with the clinico-pathological characteristics of the tumors were examined.

**Results:**

The mean age at diagnosis was 48.7 years ±11.4. Invasive ductal carcinoma was the predominant histological type (77.1%), followed by lobular invasive carcinoma (15.3%). The mean size of breast tumors was 3.5 cm ± 1.96, and 84% of our patients are diagnosed with tumors of more than 2 cm. Histological grade II tumors were the most frequent (70.4%), followed by advanced histological grade (18%). Lymph node positive tumors were observed in 64.8% of cases and 29.3% of patients had distant metastasis. Most tumors were hormone receptor-positive (73%) and 28.6% were HER2 positive. 86.1% of patients with hormone receptor-positive breast cancer were given hormone therapy, while 68.9% of patients with HER2+ breast cancer received targeted therapy with Herceptin. Luminal A was the commonest molecular subtype, followed by Luminal B, Triple Negative and HER2. The highest prevalence of premenopausal patients was observed in Triple Negative subtype (72.2%), followed by HER2 (64.1%), Luminal B (62.2%), and Luminal A (55.1%). Luminal B subtype had a poorer prognosis than Luminal A. Compared with Triple Negative, HER2 subtype tend to spread more aggressively and is associated with poorer prognosis.

**Conclusions:**

Unlike Western countries, breast cancer occurs at an earlier age and is diagnosed at a more advanced stage in Eastern Morocco. In this region, hormone receptor-positive tumors are predominant and so the majority of breast cancer patients should benefit from hormone therapy. HER2 subtype presents an aggressive tendency, suggesting the importance of anti-HER2 therapy. This study will contribute in developing appropriate screening and cancer management strategies in Eastern Morocco.

## Background

Breast cancer is a major public health problem worldwide. It is the most frequently diagnosed cancer in women worldwide, accounting for a quarter of all cancers in women [1]. In 2012, 1.67 million women were diagnosed with breast cancer [1, 2]. Based upon 2008 estimates, the incidence of breast cancer increased by more than 20%, and mortality increased by 14% [1]. Breast cancer is the most common cause of deaths from cancer in women [1].

Breast cancer is a complex disease characterized by the accumulation of multiple molecular alterations which confer to each tumor a specific phenotype [3–5]. It is a heterogeneous disease, with a variable clinical and pathological behavior and also a different prognosis and response to cancer therapies [6]. In 2000, a molecular classification of breast cancer based on gene expression profiles was proposed for the first time by Perou and his colleagues [7–11]. Then this molecular classification has been validated by several teams [7–11]. Therefore, at least four breast cancer subgroups can be identified: Luminal A and Luminal B (which are positive for the hormone receptors), HER2 (which overexpresses the *HER2* growth factor), and Basal-like (which is triple negative: estrogen receptor-negative (ER-), progesterone receptor-negative (PR-), and HER2-negative (HER2-). The identification of these breast cancer molecular subgroups provides important information on the prognosis and the response to treatment of the disease. Indeed, Luminal tumors are associated with a better prognosis compared with basal-like or HER2 tumors which have a more aggressive clinical outcome. Many studies have shown that it is possible to reproduce this molecular classification by using simple immunohistochemical tests based on the expression of *ER*, *PR*, *HER2*, *Ki-67* and other biomarkers such as high and low molecular weight cytokeratin *CK5/6* [4, 12–16]. Thus, immunohistochemical analysis was used as an alternative to determine the molecular subtypes of breast cancer and their distribution in several populations.

We have shown recently that breast cancer is the most frequent malignancy among women in Eastern Morocco [17]. However, nothing is known about the characteristics of this disease among women in Eastern Morocco. To our knowledge, only two studies on breast cancer molecular subtypes among Moroccan women were published so far. These studies were conducted in the same laboratory and during overlapping time periods. Moreover, these studies were carried out on a small sample size (366–390 cases) and they showed very discrepant results [18, 19]. Therefore, to gain further insight into breast cancer and molecular subtypes among Moroccan women, we studied the clinico-pathological and therapeutic characteristics of 2260 breast cancer cases from Eastern Morocco. Then, molecular subtypes were determined and their associations with the clinico-pathological characteristics of the tumors were examined. This study represents the largest population–based study on breast cancer among Moroccan women.

## Methods

### Setting

Eastern Morocco is located in the north east of the Kingdom of Morocco, and is the third largest region of the Kingdom [17]. The present retrospective study was based on all female breast cancer cases that were registered at the Hassan II Regional Oncology Center (ROC), since it was created in October 2005 until December 2012. During this study period, the ROC was the only health care facility for cancer management in Eastern Morocco.

### Data collection

The data were collected from patient medical records, pathology reports and admission records. A form has been used for collecting information recorded on each breast cancer case, such as the record number, name and surname of the patient, gender, place of residence, age at diagnosis, menopausal status, and clinical characteristics including tumor size, Scarff-Bloom Richardson grade, lymph-node positivity, metastasis status, immunohistochemical (IHC) markers status (ER/PR/HER2), and treatment.

### Classification of breast cancer molecular subtypes using IHC data

Tumors were classified into molecular subtypes based on IHC markers status findings [20, 21]. Thus, four subtypes were defined:

*Luminal A*:

- ER-positive, PR-positive (>20%), HER2-negative, Ki-67 < 14%.

*Luminal B*:

- ER-positive, HER2-negative, and at least one of: Ki-67 ≥ 14%, PR < 20%.
- ER-positive, HER2-positive, Any Ki-67, Any PR.

*HER2*:

- ER-negative, PR-negative, HER2-positive.

*Triple Negative*:

- ER-negative, PR-negative, HER2-negative.

### Statistical analysis

Data collection was performed on Excel. Statistical analysis was performed using SPSS software version 21.0. To evaluate the correlation between molecular subtypes and clinicopathologic variables we used Chi2 test. To assess the differences between molecular subtypes with regards to age at diagnosis and tumor size we used one-way analysis of variance (ANOVA). The results were considered significant when *p* (degree of significance) is less than 0.05, very significant when *p* < 0.01 and highly significant when *p* < 0.001.

## Results

A total of 2406 new cases of female breast cancer were registered at the ROC since it was created in October 2005 until December 2012. 27 cases were *in situ* breast cancer and were eliminated (1.2% of total) because this study was restricted to invasive breast cancer. 119 cases for which IHC data were not available were also excluded from the study. Thus, 2260 cases of invasive breast cancer were analyzed in this study. Data for age at diagnosis were available for 2220 cases. Among these, the age of patients at diagnosis ranged from 19 to 105 years with a mean age of 48.7 years ±11.4 and a median age of 48 years (Table 1).

**Table 1:** Characteristics of breast cancer patients in Eastern Morocco

Characteristics	
Age at diagnosis (*n* = 2220)	
Mean (years ± SD)	48.7 ± 11.4
Median (years)	48
Histological type (*n* = 2260)	
Invasive ductal carcinoma	77.1%
Invasive lobular carcinoma	15.3%
Medullary carcinoma	1.9%
Mucinous carcinoma	1.3%
Papillary carcinoma	0.5%
Other types	3.9%
Tumor size (*n* = 1435)	
Mean (cm ± SD)	3.5 ± 1.96
Median (cm)	3
≤ 2 cm	16%
> 2 cm	84%
Histological grade SBR (*n* = 1538)	
I	11.6%
II	70.4%
III	18%
Lymph node status (*n* = 1426)	
N0	35.2%
N+	64.8%
Metastasis status (*n* = 1311)	
M0	70.7%
M+	29.3%
IHC markers status (*n* = 2260)	
ER+ and/or PR+	73%
ER+	64.2%
PR+	66.5%
HER2+	28.6%
Use of targeted therapy among HRE2+ tumors	
Non	31.1%
Herceptin (Trastuzumab)	68.9%
Use of hormone therapy among HR+ tumors	
No	13.9%
Yes	86.1%
Therapy used among postmenopausal patients with HR+ tumor and treated with hormone therapy	
Tamoxifen	79.3%
Aromatase inhibitors	15.2%
Tamoxifen then Aromatase inhibitors	5.5%
Therapy used among premenopausal patients with HR+ tumor and treated with hormone therapy	
Tamoxifen	100%

Regarding histological tumor type, invasive ductal carcinoma was the most common histological type accounting for 77.1% of all cases, followed respectively by invasive lobular carcinoma (15.3%), medullary carcinoma (1.9%), mucinous carcinoma (1.3%), and papillary carcinoma (0.5%). 3.9% of the cases had cancers of rare histology and were grouped as other types (Table 1). The tumor size was higher than 2 cm in 84% of cases and the mean tumor size was 3.5 cm ± 1.96 (Table 1). According to the SBR grading system, the majority of tumors were grade II accounting for 70.4% of cases, followed respectively by the grade III (18%), and grade I (11.6%) (Table 1). In this study, among patients with known lymph node status, 64.8% had positive lymph-nodes, and among patients with known metastasis status, 29.3% developed distant metastases (Table 1).

Immunohistochemical data revealed that 73% of tumors were hormone receptor-positive breast cancers (ER + and / or PR +), 64.2% were estrogen receptor-positive (ER+), 66.5% were progesterone receptor-positive (PR+) and 28.6% were HER2 positive (HER2+) (Table 1). Among patients with HER2+ breast cancer, 68.9% received targeted therapy with Herceptin (Table 1). While of the cases with hormone receptor-positive breast cancer, 86.1% received hormone therapy (Table 1). Among postmenopausal women with hormone receptor-positive breast cancer and treated with hormone therapy, 79.3% of the cases received Tamoxifen, 15.2% were treated with aromatase inhibitors, and 5.5% received Tamoxifen then aromatase inhibitors (Table 1). In premenopausal patients with hormone receptor-positive breast cancer and treated with hormone therapy, 100% received Tamoxifen (Table 1).

On the basis of the immunohistochemical markers status, the breast cancer cases were classified into four breast cancer molecular subtypes. The distribution of these subgroups is shown in Table 2. Luminal A was the most common subtype (61.1%), followed respectively by Luminal B (16.1%), Triple Negative (14.2%), and then HER2 (8.6%). The characteristics of these breast cancer molecular subtypes in Eastern Morocco are presented in Table 2. The molecular subtypes differed significantly by age at diagnosis (*p*-value = 0.038) (Table 2). The molecular subtypes have also shown a very significant difference according to menopause status (p-value = 0.004) (Table 2). Indeed, among Triple Negative tumors, 72.2% were premenopausal, while among Luminal A tumors only 55.1% were premenopausal (Table 2).

**Table 2:** Characteristics of breast cancer molecular subtypes in Eastern Morocco

Characteristics	Luminal A	Luminal B	HER2	Triple Negative	*P-value*
	(*N* = 1381)	(*N* = 364)	(*N* = 195)	(*N* = 320)	
Percentage of subtypes	61.1%	16.1%	8.6%	14.2%	
Median age (years)	48	47	49	45	0.038
Menopausal status					
Premenopausal	55.1%	62.2%	64.1%	72.2%	0.004
Postmenopausal	44.9%	37.8%	35.9%	27.8%	
Histological type					
Invasive ductal carcinoma	80,2%	85,7%	81,4%	80%	0.02
Invasive lobular carcinoma	15,5%	8,5%	14%	6,3%	
Medullary carcinoma	0,8%	0,5%	0%	5%	
Mucinous carcinoma	1,4%	2,1%	1,2%	0,6%	
Papillary carcinoma	0,9%	0,5%	0%	0%	
Other types	1,2%	2,6%	3,5%	8,1%	
Tumor size					
Mean size cm ± SD	3.38 ± 2.1	3,42 ± 2.2	3.80 ± 2.1	3.78 ± 1.9	0.097
< 2.0	17.3%	16.7%	13.8%	13.2%	0.005
2.0 – 5.0	60.4%	56.3%	51.6%	52.8%	
> 5.0	22.3%	27.1%	34.6%	34%	
Histological grade (SBR)					
I	16.6%	8,10%	4.2%	5.2%	<0.0001
II	71.2%	74.5%	70.4%	64.6%	
III	12.2%	17.4%	25.4%	30.2%	
Vascular emboli status					
Negative	32.9%	27%	28%	38.1%	0.209
Positive	67.1%	73%	72%	61.9%	
Lymph node status					
N0	36.1%	32.9%	28.6%	40.8%	0.114
N+	63.9%	67.1%	71.4%	59.2%	
Metastasis status					
M0	72.4%	74.5%	58.1%	69.9%	0.059
M+	27.6%	25.5%	41.9%	30.1%	

The molecular subtypes differed significantly by histological type (Fisher exact test p-value = 0.02) (Table 2). The Triple Negative subtype showed the highest prevalence of medullary carcinoma histological type, 5% versus 0.8% Luminal A, 0.5% Luminal B and 0% HER2 (Table 2). Furthermore, Triple Negative subtype showed the lowest prevalence of invasive lobular carcinoma and mucinous carcinoma histological types compared with the other subtypes (Table 2).

The molecular subtypes did not differ by mean size of tumors at diagnosis **(**p-value = 0.097) (Table 2). However, we found significant difference by tumor size among breast cancer subtypes when stratified into groups: < 2.0 cm, 2.0 – 5.0 cm, and > 5.0 cm **(**p-value **=** 0.005) (Table 2). Regarding the histological grade, the molecular subtypes differed significantly (p-value <0.0001) (Table 2). The HER2 and Triple Negative subtypes had the highest percentage of advanced histological grade (grade III) in comparison with the Luminal subtypes (Table 2).

All molecular subtypes had a high percentage of vascular emboli, with 73%, 72%, 67.1%, and 61.9% for Luminal B, HER2, Luminal A, and Triple Negative respectively (Table 2). However, the molecular subtypes did not differ significantly with regards to vascular emboli status (p-value = 0.209). Nevertheless, in comparison to Triple Negative, patients with HER2 subtype had a higher percentage of vascular emboli. Regarding lymph node status at time of diagnosis, the molecular subtypes did not differ significantly (p-value = 0.114) (Table 2). HER2 subtype had the highest prevalence of positive lymph nodes (71.4%), followed respectively by Luminal B (67.1%), Luminal A (63.9%) and finally Triple negative (59.2%) (Table 2). Concerning the metastasis status, the molecular subtypes did not differ significantly (p-value = 0.059) (Table 2). HER2 subtype showed with the highest level of metastases, 41.9% versus 27.6% Luminal A and 25.5% Luminal B (Table 2). Moreover, in comparison to Triple Negative, patients with HER2 subtype had a higher level of metastases.

## Discussion

Breast cancer is the main cause of death among women worldwide [22, 23]. Breast cancer is a complex and heterogeneous disease associated with clinical, pathological and biological factors that are variable from one population to another [4]. These prognostic factors are essential for the management of breast cancer. Moreover, molecular classification of breast cancer is now an important tool to guide patient management. Therefore, to gain further insight into breast cancer and molecular subtypes among Moroccan women, we analyzed 2260 breast cancer cases from Eastern Morocco. Thus, to our knowledge this paper represents the first study in Morocco on breast cancer using a large series of patients.

### Age at diagnosis of breast cancer in Eastern Morocco

Our study revealed that in Eastern Morocco the age at the time of breast cancer diagnosis was between 19 and 105 years, with a mean age of 48.7 years ±11.4 and a median age of 48 years. This result is similar to those reported in other North African countries, Iraq, Sub-Saharan Africa, Korea, China, and Taiwan [18, 24–32]. However, in Western countries and other Arab and Asian countries breast cancer occurs at an older age [26, 27, 33–36]. The age at diagnosis represents an important prognostic factor since tumors diagnosed at younger ages are generally more aggressive and/or less responsive to treatment [37–39]. Multiple factors may be involved in the age at diagnosis, including the structure of the population, genetic and environmental factors. In Eastern Morocco, this young age at diagnosis of breast cancer may be simply due to the young population in this region [40]. Further studies are needed to confirm the involvement of these factors in the occurrence of cancer at a younger age in Eastern Morocco.

### Histological types of breast cancer in Eastern Morocco

In our study, invasive ductal carcinoma was the predominant histological type, which is similar to most previous studies on breast cancer [13, 28–30, 41–47]. Lobular invasive carcinoma was the second most common histological type accounting for 15.3% of all cases. This level of lobular invasive carcinoma in our study is higher compared to what has been reported in Fez (Morocco) (4%), Egypt (9%), Saudi Arabia (3%) and Iraq (3.9%) [18, 26, 36, 48].

### Clinico-pathological features of breast cancer in eastern Morocco

In Western countries the majority of breast tumors measure less than 2 cm, reflecting the early detection of the disease [33, 36, 49–51]. In our series, the mean size of breast tumors was 3.5 cm ± 1.96, which is similar to those reported in Fez (Morocco), and in other Arab countries [18, 24, 36]. However, the percentage of tumors that measure larger than 2 cm was higher in our study as compared to those observed in other Arab and Asian studies [27, 35, 52–55]. In this study, we found a high percentage of histological grade II tumors (70.4%) and an intermediate level of advanced histological grade (18%). Such a finding is far from that reported in Fez, where a lower percentage of grade II tumors (54.7%), and a higher level of histological grade III (30.7%) were observed [33]. Lymph node positive tumors were observed in 64.8% of cases and 29.3% of patients had distant metastasis. The previous Fez study showed a lower percentage of positive lymph nodes (53%) and distant metastasis (17.5%) [33]. In developed countries, the majority of patients have a negative lymph node status [13, 50, 56, 57]. This is due to efficient breast cancer screening programs, which contribute to the early diagnosis of breast cancer [58, 59]. Therefore, the high rate of metastasis observed in this study is probably due to late diagnosis of breast cancer and highlights the necessity of establishing an effective breast cancer screening program in Eastern Morocco.

### Hormone receptor status and hormone therapy in Eastern Morocco

Our study revealed that in Eastern Morocco, 64.2% of tumors were ER+. Such a finding is similar to those observed in other North African countries, Saudi Arabia, China and the United States [13, 28, 41, 60–65]. Regarding the PR status, we found that the majority of tumors were PR+ (66.5%). This result is similar to those reported in Fez, Saudi Arabia, but higher than the figure reported in other North African countries [13, 28, 41, 60–65]. Hormone Receptor (HR) status has important implications for breast cancer prognosis and management. Indeed, hormone receptor-positive tumors are usually associated with a favourable prognosis and can be given endocrine therapy. Thus, the assessment of HR using IHC assays is essential before any treatment. However, in low-income countries, HR status is not performed for patients of low-resource. In such countries, if the prevalence of hormone receptor-positive tumors is very high it is possible to apply hormone therapy even among patients in whom HR status has not been conducted [66, 67]. Regarding hormone therapy, a number of studies have previously shown that treatment of ER+ breast cancer with Tamoxifen significantly reduces the risk of recurrence and mortality [68, 69]. Tamoxifen is a drug that works by preventing the binding of estrogen to tumor cells, and is effective in both postmenopausal and premenopausal patients. Aromatase inhibitors (AIs) are another class of drugs that are used to treat postmenopausal patients with hormone receptor-positive breast cancer. Several studies have recently shown that treatment of hormone receptor-positive breast cancer in postmenopausal women with either aromatase inhibitors (AIs) alone or after Tamoxifen gives a clear advantage in comparison with treatment using Tamoxifen alone [70, 71].

In this study, we found that 86.1% of patients with HR+ breast cancer were given hormone therapy. Tamoxifen was used to treat the majority of our premenopausal (100% of cases) and postmenopausal (79.3% of cases) patients. Only 15.2% of postmenopausal patients received AIs alone, and 5.5% of cases were treated with AIs after Tamoxifen. Together, these population-based estimates of the HR status in Eastern Morocco can aid treatment decisions. These data indicate that since in Eastern Morocco the majority of patients showed hormone receptor-positive breast cancer, hormone therapy should be applied even for patients in whom HR status has not been conducted. Moreover, therapy with AIs should be used more often in the case of postmenopausal patients with hormone receptor-positive breast cancer.

### HER2 status and targeted therapy in Eastern Morocco

In this study, we found that 28.6% of tumors were HER2+. This result is similar to those reported in other North African countries, Saudi Arabia, but higher than the figure reported in Europe, the United States and most countries in Sub-Saharan Africa [13, 18, 28, 30, 60, 62, 63, 65, 72, 73]. Women whose breast cancers test positive for HER2 can be given targeted therapy. Herceptin is a monoclonal antibody that directly targets the HER2 protein. In 2006, this drug has been approved by the FDA (US Food and Drug Administration) for the treatment of all HER2+ breast cancers [74]. It is currently the standard treatment for HER2+ tumor, and it is used in combination with other treatment [75–77]. In this study, we found that only 68.9% of patients with HER2+ breast cancer received targeted therapy with Herceptin. Together, these data indicate that in Eastern Morocco, tumors overexpressing HER2 protein are frequent and thereby should benefit from targeted therapy. Thus, affordable treatment for HER2-positive tumors with targeted therapy aimed at HER2 is needed in Eastern Morocco.

### Distribution of molecular breast cancer subtypes in Eastern Morocco

As mentioned earlier, only two studies have been reported on molecular breast cancer subtypes among Moroccan women [18]. Although these studies were conducted in the same laboratory and during overlapping time periods these showed very discrepant results: Luminal B was the most frequent molecular subtype in El Fatemi et al., (2012), while Luminal A was the predominant subtype in Bennis et al., (2012). In this study, Luminal A was the commonest molecular subtype with 61.1%. This result is similar to that reported in white American population (62%) (Table 3) [78]. However, a lower frequency of Luminal A has been reported in other North African countries (30.5–53.6%), Sub-Saharan countries (25.6–53.5%), China (48.6%), black, Hispanic and Asian American (47–55%) (Table 3) [42, 45, 60, 62, 65, 79–81]. A higher prevalence of Luminal A than our series has been reported in European countries (68.7–75%) (Table 3) [45, 60, 62, 65, 79, 80]. The proportion of Luminal B in this study (16.1%) is similar to that found in other North African countries (13.4–19.7%), and China (16.7%) (Table 3) [45, 60, 62, 65, 79, 80]. However, a lower frequency of Luminal B than our series has been reported in Sub-Saharan African countries (12.2–14.6%), European countries (6–12.5%), and American population (9–12%) (Table 3) [45, 60, 62, 65, 79, 80].

**Table 3:** Distribution of breast cancer molecular subtypes in different countries

Country	Luminal A	Luminal B	HER2	Triple Negative	N.	R.
North Africa						
Eastern Morocco this study	61.1	16.1	8.6	14.2	2260	
Fez-Morocco	53.6	16.4	12.6	17	366	[18]
Fez-Morocco	30.5	41.8	9.2	13.6	390	[19]
Tunisia	51.5	16	14.5	18	194	[31]
Tunisia	50.7	13.4	13.4	22.5	966	[63]
Algeria	50.6	19.7	8.9	20.8	3014	[30]
Sub-Saharan Africa						
Mali	29.2	13.2	4.4	46.5	114	[73]
Ghana	25.6	12.2	12.8	49.4	165	[81]
South Africa	53.5	14.6	11.4	20.5	1218	[42]
Europe						
Poland	69.0	6.0	8.0	12.0	804	[79]
Italy	75.0	7.0	3.0	12.0	138	[60]
Spain	68.7	12.5	7	11.8	2771	[45]
USA						
White	62	9	4	9	50248	[78]
Black	47	11	6	20	4848	[78]
Hispanic	52	11	7	13	12700	[78]
Asian	55	12	8	9	8441	[78]
Asia						
China	48.6	16.7	13.7	12.9	2791	[41]

N.: number of patients analyzed; R.: references

The HER2 subtype in this study accounted for 8.6% of all breast cancer cases, which is similar to that reported in Algeria (8.9%), but lower than in other North African countries (9.2–14.5%), and China (13.7%) (Table 3) [45, 60, 62, 65, 79, 80]. A lower prevalence of HER2 than our series has been reported in American population (4–8%) and European countries (3–8%) (Table 3) [45, 60, 62, 65, 79, 80]. The prevalence of Triple Negative in this study (14.2%) is slightly higher than in European countries (11.8–12%), China (12.9%), and the white, Hispanic and Asian ethnic groups of the US population (9–13%) (Table 3) [45, 60, 62, 65, 79, 80]. A higher proportion of Triple Negative than our series has been reported in other North African countries (18–22.5%), Sub-Saharan African patients (20.5–49.4%) and black American (20%) (Table 3) [45, 60, 62, 65, 79, 80].

Together, these data suggest that the distribution of molecular subtypes among Moroccan women is similar to that reported in most studies over the world, with Luminal A being the most common subtype, followed by Luminal B, Triple Negative and HER2. The most likely explanation for the discrepancies among the two previous studies carried out in Fez in Morocco seems to be differences in sensitivity of the method of HR status assessment and/or in the criteria for classifying molecular breast cancer subtypes.

### Molecular breast cancer subtypes associations with clinico-pathological factors

The molecular subtype showed no statically significant difference according to mean size of tumors at diagnosis, vascular emboli status, lymph node status or metastasis status. However, molecular subtype differed significantly with regards to age at diagnosis. We found that the Triple Negative subtype was associated with a younger age at diagnosis compared with the other subtypes, similar to previous reports on different populations [13, 42, 63, 82, 83]. The molecular subtypes have also shown a very significant difference by menopause status. The highest prevalence of premenopausal patients was observed in Triple Negative subtype, while Luminal A was associated with the lowest prevalence of premenopausal patients, consistent with previous studies [13, 84–86].

Regarding the histological grade, a significant difference was noted among the molecular subtypes. Luminal A was associated with the lowest proportion of histological grade III, association that has been noted by other studies [4, 13, 41, 42, 45, 83, 85, 87]. Luminal A had also the lowest proportion of tumors with diameter larger than 5 cm. These findings are consistent with previous studies that have shown that Luminal A tumors tend to be slow-growing and are associated with a good prognosis [4, 88]. In this study, Luminal A subtype had a better prognosis than Luminal B subtype. Indeed, in comparison with Luminal A, Luminal B had a higher percentage of tumors with a large diameter (>5.0 cm), a larger proportion of histological grade III, and a higher percentage of vascular emboli and lymph node involvement. These data are consistent with previous studies [89, 90].

In comparison with the other subtype, Triple Negative was associated with the highest proportion of histological grade III, the lowest median age at diagnosis, and the highest prevalence of premenopausal patients. These are consistent with several previous studies that have shown that Triple Negative is more common in premenopausal women and it is an aggressive subtype [13]. In this study, we found also that Triple Negative subtype had the lowest prevalence of positive lymph nodes and the lowest proportion of positive vascular emboli. These results are in accordance with previous studies that have shown that Triple Negative is less likely to demonstrate positive lymph nodes and positive vascular emboli when compared with the other subtypes [91, 92]. In our series, Triple Negative subtype showed the highest prevalence of medullary carcinoma. Previous studies have shown that this histological type is characterized by a high histological grade and rare lymph node metastasis [91, 92]. Interestingly, these clinico-pathologic features of medullary carcinoma are consistent with those observed for Triple Negative subtype in this study.

In comparison with Triple Negative subtype, HER2 had similar percentage of tumors with a large diameter (>5 cm) (34.6% versus 34%), but a higher percentage of lymph-node positive tumors (71.4% versus 59.2%), a larger proportion of positive vascular emboli (72% versus 61.9%), and a higher level of distant metastases (41.9% versus 30.1%). Together, these data indicate that in Eastern Morocco, HER2 breast cancers tend to grow and spread more aggressively than Triple Negative subtype breast cancers and are associated with poorer prognosis, and thus highlight the need to the use of targeted therapies for HER2 cancers in this region.

## Conclusions

This study revealed alarming features of breast cancer in Eastern Morocco, with a large tumor size, a high histological grade, a significant percentage of vascular emboli, a large percentage of lymph node involvement, and distant metastases. Thus, our finding highlights the importance to strengthen the breast cancer screening program in Eastern Morocco. Moreover, the observed occurrence of breast cancer at a younger age in Eastern Morocco justifies the need to establish a screening program at an earlier age in this region. Our results indicate that in Eastern Morocco, hormone receptor-positive and HER2+ tumors are predominant and so the majority of breast cancer cases in this population may benefit from hormone therapy and/or targeted therapy. Molecular subtype analyses revealed that Luminal A was the commonest molecular subtype, followed by Luminal B, Triple Negative and HER2. Luminal B subtype in our study had a poorer prognosis than Luminal group A. Compared with the Triple Negative subtype, the HER2 subtype in our study tends to spread more aggressively and has a poorer prognosis, suggesting the importance of anti-HER2 therapy. This study will contribute in developing appropriate screening and cancer management strategies in Eastern Morocco.
